# A successful treatment of esophagomediastinal fistula following endoscopic submucosal dissection: tube-in-tube endoscopic vacuum therapy

**DOI:** 10.1055/a-2723-1617

**Published:** 2025-11-19

**Authors:** Xiaoyan Zhang, Dandan Zhao, Tianxu Jia, Yinglin Niu, Huihong Zhai

**Affiliations:** 1 71044 Department of Gastroenterology, Xuanwu Hospital Capital Medical University, Beijing, China; 2 26455 Department of Gastroenterology, Capital Medical University Affiliated Beijing Friendship Hospital, Beijing, China

A 69-year-old female patient underwent endoscopic submucosal dissection (ESD) to treat circumferential, moderately differentiated squamous cell carcinoma in the middle esophagus. She developed cough with sputum 4 days after ESD. Computed tomography (CT) revealed an esophagomediastinal fistula with associated abscess cavity formation. Esophagogastroduodenoscopy (EGD) showed an esophageal perforation (10 mm ×  8 mm) at the resection site ( Fig. 1 ).

**Fig. 1:**
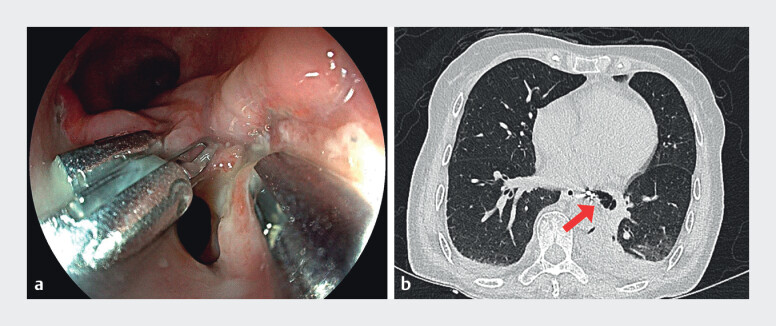
Esophagomediastinal fistula following endoscopic submucosal dissection. **a** The orifice of the fistula. **b** Computed tomogram showed an esophagomediastinal fistula (red arrow).

Considering the minimal fistula orifice and established abscess cavity formation, we decided to utilize tube-in-tube endoscopic vacuum therapy (TT-EVT) as the definitive treatment approach 1 2 . We assembled the tube-in-tube device by mounting a 16-Fr Levin nasogastric tube (Link-02-1; Beijing L&Z Medical, Beijing, China) over a transendoscopic enteral tube (outer diameter: 2.7 mm, FMT-DT-N-27/1350; FMT Medical, Nanjing, China). Precision coaxial perforations were created in both tubes using a 16-G needle. Then, we threaded a nylon suture through these perforations and tied it to form a coil for traction ( Fig. 2 ). The nasogastric tube functioned as the outer tube, providing structural support as well as preventing tissue aspiration or clogging. The transendoscopic enteral tube, which served as the inner tube, was connected to the wall-mounted vacuum suction pump using a suction connection tube. The system delivered continuous negative pressure at 300 mmHg (0.04 MPa), with twice-daily irrigation performed using normal saline solution (0.9% sodium chloride). We advanced the tube-in-tube drain by grasping the traction coil and positioned it inside the mediastinal abscess under endoscopic guidance ( Video 1 ).

**Fig. 2:**
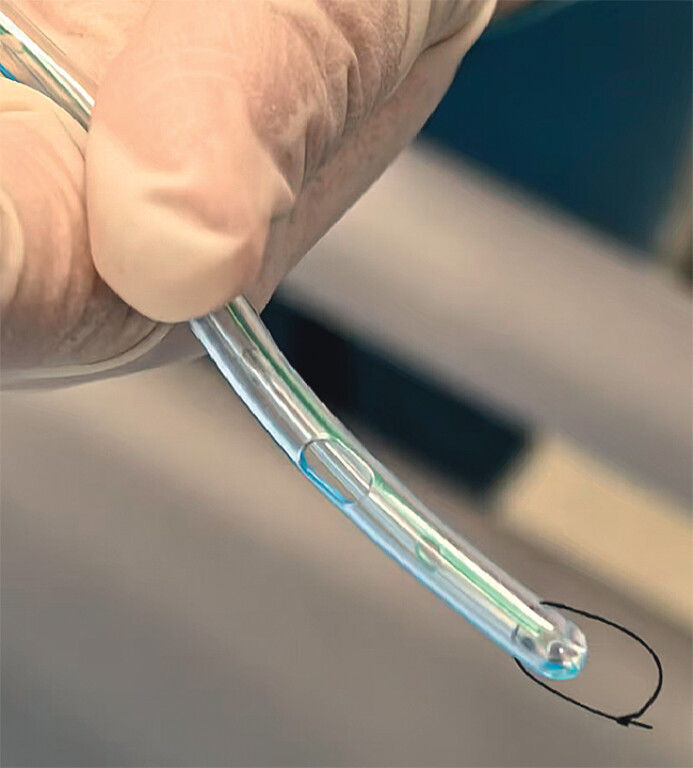
The tube-in-tube drain.

Tube-in-tube endoscopic vacuum therapy to treat esophagomediastinal fistula following endoscopic submucosal dissection.Video 1

We pulled out the drain 1 cm every 2 or 3 days. Follow-up endoscopic examinations showed abscess cavity size reduction associated with granulation tissue formation. On day 24, after the device placement, follow-up EGD revealed significant reduction of the fistula orifice to approximately 1 mm in diameter. No purulent discharge was observed, prompting tube-in-tube drain removal. 22 days after tube removal, the mucosa at the fistula site has completely healed. Three-month follow-up CT showed complete resolution of the abscess ( Fig. 3 ).

**Fig. 3:**
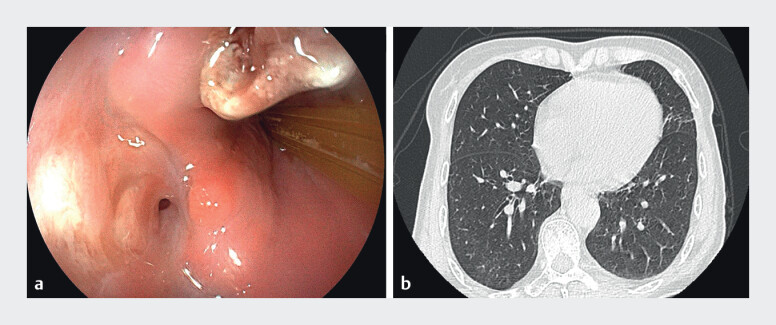
Residual fistula after drainage tube extraction. **a** The fistula orifice following drain removal. **b** Computed tomogram revealed complete resolution of the abscess cavity.

Tube-in-tube endoscopic vacuum therapy (TT-EVT) was first described in 2021 for the treatment of upper gastrointestinal leaks and perforations 3 . As an EVT modification, TT-EVT enables effective drainage and irrigation and is easy to make using readily available materials. We successfully managed this post-ESD esophagomediastinal fistula using the TT-EVT approach without using other endoscopic methods. Our report offers practical evidence for similar cases.

Endoscopy_UCTN_Code_TTT_1AR_2AZ

## Notes

Endoscopy E-Videos https://eref.thieme.de/e-videos
*E-Videos* is an open access online section of the journal *Endoscopy* , reporting on interesting cases and new techniques in gastroenterological endoscopy. All papers include a high-quality video and are published with a Creative Commons CC-BY license. Endoscopy E-Videos qualify for HINARI discounts and waivers and eligibility is automatically checked during the submission process. We grant 100% waivers to articles whose corresponding authors are based in Group A countries and 50% waivers to those who are based in Group B countries as classified by Research4Life (see: https://www.research4life.org/access/eligibility/ ). This section has its own submission website at https://mc.manuscriptcentral.com/e-videos .
